# Control of visceral leishmaniasis in Brazil: recommendations from Brasileish

**DOI:** 10.1186/1756-3305-6-8

**Published:** 2013-01-11

**Authors:** Vitor Márcio Ribeiro, Sydnei Magno da Silva, Ingrid Menz, Paulo Tabanez, Fábio dos Santos Nogueira, Manfredo Werkhaüser, André Luis S da Fonseca, Filipe Dantas-Torres

**Affiliations:** 1 Escola de Veterinária, Pontifícia Universidade Católica de Minas Gerais, Angola, Brazil; 2 Dep. de Imunologia, Microbiologia e Parasitologia, Instituto de Ciências Biomédicas, Universidade Federal de Uberlândia, Uberlândia, Brazil; 3 Médica Veterinária Autônoma, Campinas, Brazil; 4 Médico Veterinário Autônomo, Brasília, Brazil; 5 Médico Veterinário Autônoma, Andradina, , , Brazil; 6 Médico Veterinário Autônoma, Belo Horizonte, , , Brazil; 7 Dep. de Patologia, Universidade Federal Mato Grosso do Sul, Campo Grande, Brazil; 8 Dep. de Imunologia, Centro de Pesquisas Aggeu Magalhães, Fiocruz, Recife, Brazil; 9 Dip. di Medicina Veterinaria, Università degli Studi di Bari, Valenzano, Italy

## Abstract

On 26 October 2012, veterinary medicine clinicians and researchers, members of Brasileish - Study Group about Animal Leishmaniasis - met at the Regional Council of Veterinary Medicine of Minas Gerais, in the city Belo Horizonte, in order to discuss many aspects of the situation of canine visceral leishmaniasis (CVL) in Brazil. In the face of recent national and international scientific evidence, we, the members of Brasileish, have elaborated some recommendations for the management and control of CVL in Brazil.

## Background

On the 26th of October 2012, veterinary medicine clinicians and researchers, members of *Brasileish –A Study Group about Animal Leishmaniasis* - met at the Regional Council of Veterinary Medicine of Minas Gerais, in the city Belo Horizonte, in order to discuss many aspects of the situation regarding canine visceral leishmaniasis (CVL) in Brazil. Among these, questions concerning the limitations of the serological tests currently recommended in the country, the recommendations for canine elimination as a control measure, the limitation of the treatment with drugs not used in human treatment and not registered with the Ministry of Agriculture, Livestock and Food Supply and the use of canine vaccination as a control measure were also dealt with.

This meeting preceded the IX International Symposium on Canine Visceral Leishmaniasis held on 27-28 October 2012 at the School of Veterinary Medicine of the Federal University of Minas Gerais. At this event, various aspects of CVL, from pathogenesis to legal questions related to its control, were discussed.

In the face of recent national and international scientific evidence [1-7], and on the basis of discussions held at these two meetings, we, the members of Brasileish, have developed the following recommendations for the management and control of CVL in Brazil.

## Recommendations

### Health education

Implementing health education measures in endemic areas. These activities should be carried out jointly by health officials, who should be properly trained to inform the population about the principal measures for control of leishmaniasis, including of individual protection (use of sand fly-proof nets, netting for windows and doors, avoiding outdoor activities at twilight and at night, among others). These measures should be permanent and carried out with district associations and community leaders. Domiciliary visits, discussions in public and private schools with the aim of educating the population about responsible ownership, animal welfare and also aspects related to prevention of visceral leishmaniasis in dogs and in humans are pivotal.

### Control of the canine population

Individual registration of domestic and semi-domestic dogs by means of microchips, so as to obtain better control of the canine population in each community and so that every owner is made responsible for their pets. Canine vaccination against zoonoses, such as rabies, leptospirosis and visceral leishmaniasis, should be promoted by means of public vaccination campaigns associated with measures to control helminthic infestations, fleas, lice, and ticks. The creation of public veterinary hospitals for the treatment of animals originating from poor communities should be included in the agenda of priorities for animal health care and consequent control of zoonoses. These hospitals should provide veterinary care and sterilization procedures for dogs and cats.

### Diagnosis and treatment of dogs with CVL

The diagnosis of CVL should be made by a professional veterinarian on the basis of compatible clinical signs and parasitological, serological and/or molecular confirmation. Standardization of serological tests to reduce the possibility of cross-reactions (false positives), is essential. Seropositive dogs in the indirect immunofluorescence assay at a dilution of 1:40 should be considered suspect and should undergo other tests. A titre four times higher than the cut-off point (1:40) should be considered diagnostic. In this way only positive results in IFAT at dilutions ≥ 1:160, may be considered true positive. In areas where *Leishmania braziliensis* or other *Leishmania* species are endemic, confirmation of the etiological diagnosis is essential in order to avoid the elimination of dogs that are not infected by *Leishmania infantum* (= *Leishmania chagasi*). Owners of infected dogs must be guaranteed the right to choose between euthanasia and responsible treatment of their animals. When the option is euthanasia, it should be carried out in accordance with ethical principles and by a veterinarian. When the option is treatment, it should be carried out with protocols that produce improvement or clinical cure in the animal and a reduction in the parasite burden, which should be evaluated by means of clinical and laboratory investigations.

### Combating the vector

Control of the vector should be carried out by means of environmental management (construction of houses on barren areas, cleaning and reorganization of peridomiciliary areas), adoption of individual protective measures (netting for doors and windows, use of bed nets impregnated with insecticides in high-risk areas) and the use of insecticides in the environment in special situations (risk of epidemics, intradomiciliary transmission). The use of insecticides (collars, pipettes, sprays) on dogs is essential and must be encouraged by the government in poor areas.

The situation regarding visceral leishmaniasis in Brazil reflects the failure of the National Program for Control of Visceral Leishmaniasis. Established over 50 years ago, this program needs to be completely redesigned in the face of new scientific knowledge and new tools that are available for the control of this disease. Annual meetings should be held concerning the current situation of leishmaniasis in Brazil, involving participation of both doctors and veterinarians, whether clinicians or health officials and other agents, to promote public health. It is very important that Brazil should adopt the concepts of “One Health” in the preparation of measures for control of zoonoses such as leishmaniasis. Every management and control measure must be backed by scientific data and/or by evidence-based medicine. In this sense, searches for new diagnostic methods, treatment protocols and tools for control and prevention (vaccination and vector control) must be encouraged, financed by public development bodies, and prioritized by the competent authorities and ethics committees. By means of education and efficacious measures it will be possible to reduce the impact of leishmaniasis in Brazil.

Finally, we highlight the fact that, as emphasized more than ten years ago by Doctor Carlos Henrique Nery Costa, president of the Brazilian Society of Tropical Medicine, and Doctor João Batista Furtado Vieira, current manager of General Coordination of Leprosy and Diseases in the Process of Elimination at the Ministry of Health, the control of leishmaniasis in Brazil must change [8]. Investment is needed in monitoring, health education, and quality of life of at-risk populations.

## Competing interests

The authors declare that they have no competing interests.

N.B. This text has been published previously in Portuguese (Ribeiro VM, Silva SM, Menz I, Tabanez P, Nogueira FS, Werkhaüser M, Fonseca ALS, Dantas-Torres F: **Controle da leishmaniose visceral no Brasil: recomendações do Brasileish.***Clínica Veterinária* 2012, **17**(101):28–29).

## Authors’ contributions

All authors contributed equally to this work. All authors read and approved the final manuscript.

## References

[B1] Dantas-Torres F Solano-Gallego L Baneth G Ribeiro VM de Paiva-Cavalcanti M Otranto D Canine leishmaniosis in the Old and New Worlds: unveiled similarities and differences Trends Parasitol 2012 28 12 531 538 10.1016/j.pt.2012.08.007 22995719

[B2] Solano-Gallego L Miró G Koutinas A Cardoso L Pennisi MG Ferrer L Bourdeau P Oliva G Baneth G The LeishVet Group LeishVet guidelines for the practical management of canine leishmaniosis Parasit Vectors 2011 4 86 10.1186/1756-3305-4-86 21599936 PMC3125381

[B3] World Health Organization Control of the Leishmaniasis 2010 WHO (Technical Report Series 949), Geneva 104

[B4] Costa CH How effective is dog culling in controlling zoonotic visceral leishmaniasis? A critical evaluation of the science, politics and ethics behind this public health policy Rev Soc Bras Med Trop 2011 44 2 232 242 10.1590/S0037-86822011005000014 21468480

[B5] Passantino A Russo M Coluccio P Canine leishmaniosis and euthanasia in Italy: a critical legal-ethical analysis Rev Sci Tech 2010 29 3 537 548 21309453

[B6] Quinnell RJ Courtenay O Transmission, reservoir hosts and control of zoonotic visceral leishmaniasis Parasitology 2009 136 14 1915 1934 10.1017/S0031182009991156 19835643

[B7] Romero GA Boelaert M Control of visceral leishmaniasis in Latin America: A systematic review PLoS Negl Trop Dis 2010 4 1 e584 10.1371/journal.pntd.0000584 20098726 PMC2808217

[B8] Costa CH Vieira JB Changes in the control program of visceral leishmaniasis in Brazil Rev Soc Bras Med Trop 2001 34 2 223 228 11391448 10.1590/s0037-86822001000200013

